# Supplementary material for: Polymerization Kinetics of Acrylic Photopolymer Loaded with Graphene-Based Nanomaterials for Additive Manufacturing
Source: Nanomaterials (Basel). 2022 Dec 19;12(24):4498. doi: 10.3390/nano12244498 (PMC9783852; doi:10.3390/nano12244498)
Supplement: Supplementary file 1 [file nanomaterials-12-04498-s001.zip › nanomaterials-2034991-supplementary.pdf]

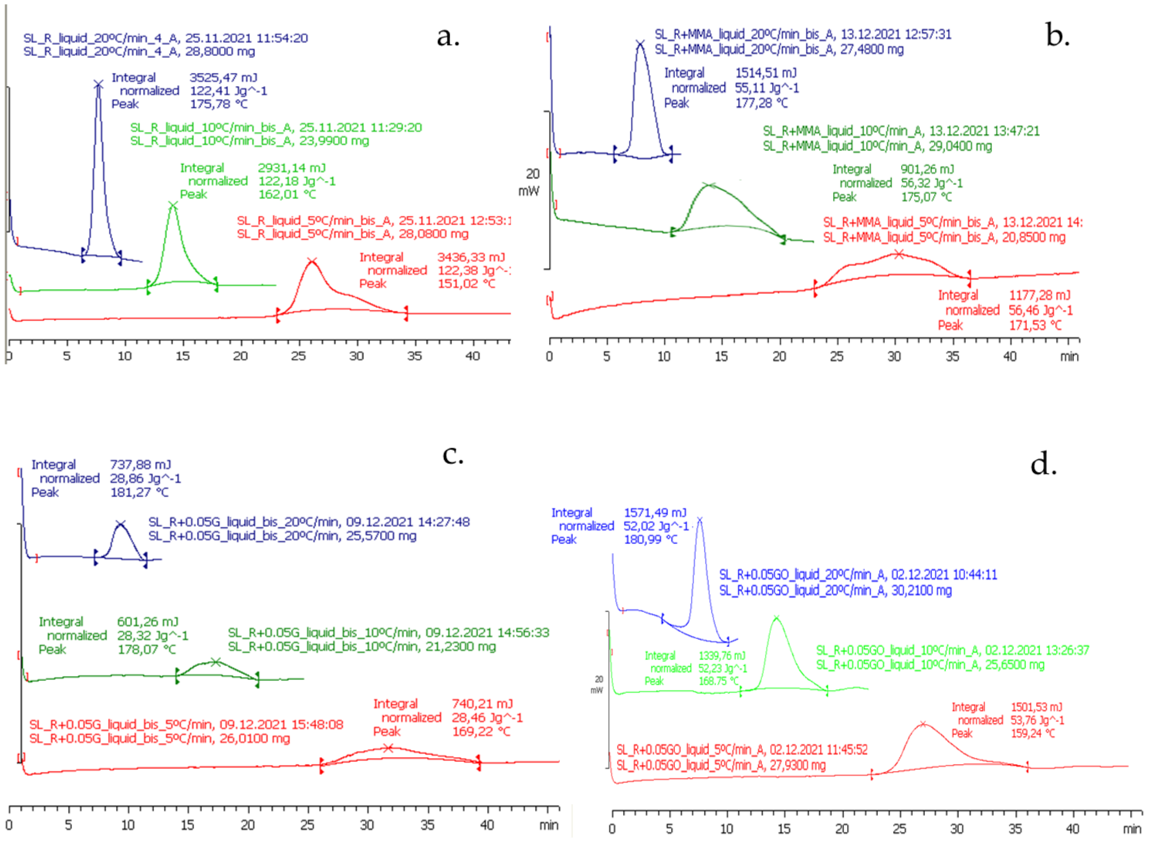

Figure S1. Thermograms of thermally-triggered polymerization of R (a), R’ (b), R’+G (c) and R’+GO (d)

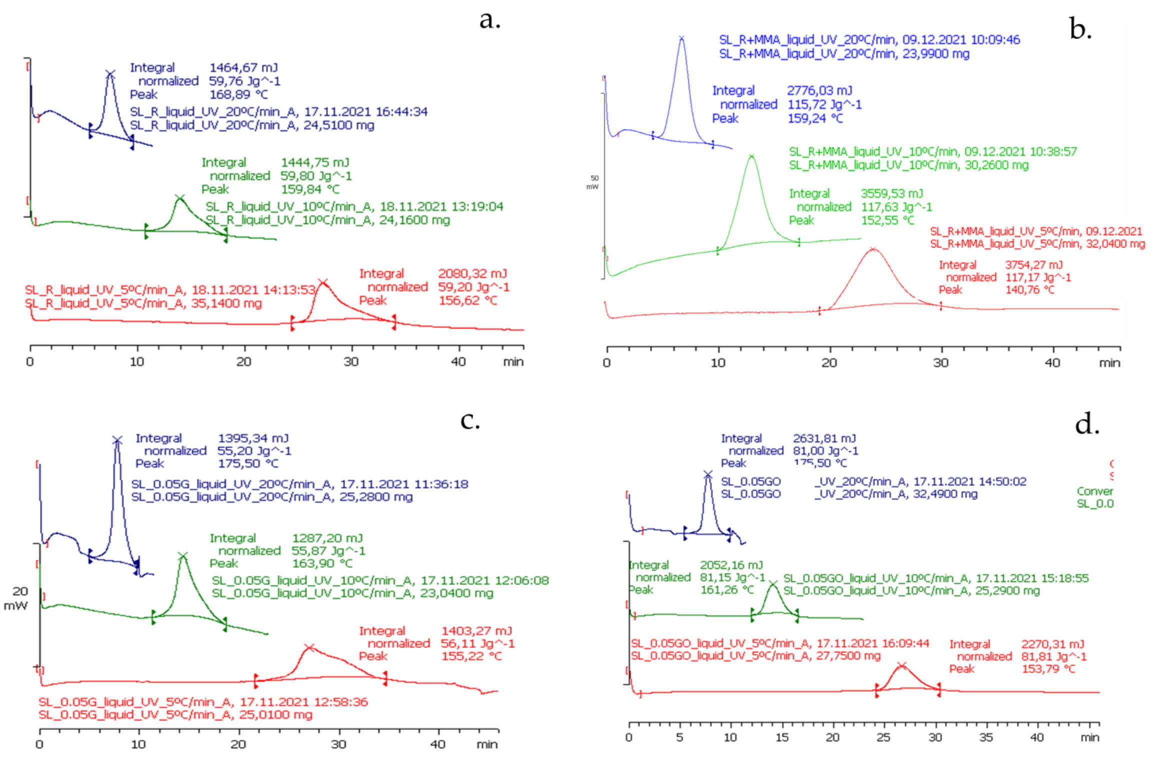

Figure S2. Thermograms of UV-triggered polymerization of R (a), R’ (b), R’+G (c) and R’+GO (d)

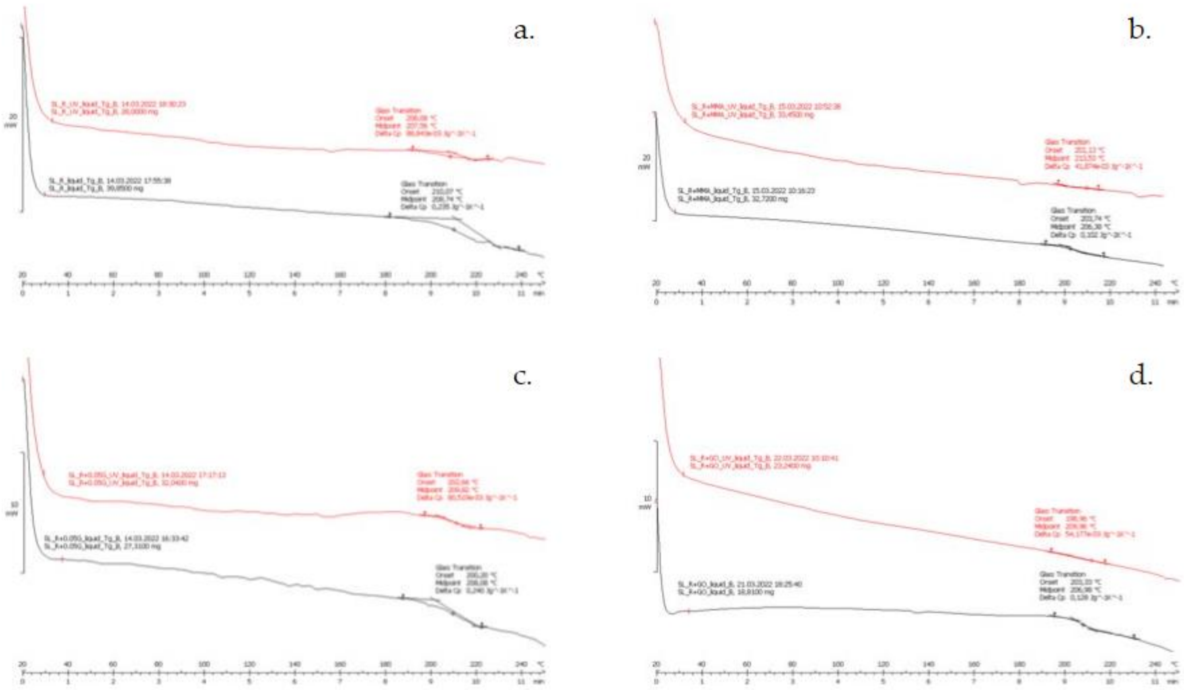

Figure S3. Second scan of UV- (red) and thermally- (black) triggered polymerized samples
